# Contribution of Fc-dependent cell-mediated activity of a vestigial esterase-targeting antibody against H5N6 virus infection

**DOI:** 10.1080/22221751.2019.1708215

**Published:** 2020-01-06

**Authors:** Zhiqiang Zheng, Su Hui Catherine Teo, Suganya Cheyyatraivendran Arularasu, Zhehao Liu, Nur Khairiah Mohd-Ismail, Chee Keng Mok, Chee Bing Ong, Justin Jang-hann Chu, Yee-Joo Tan

**Affiliations:** a Department of Microbiology and Immunology, Yong Loo Lin School of Medicine, National University Health System (NUHS), National University of Singapore, Singapore, Singapore; b Institute of Molecular and Cell Biology, Agency for Science, Technology and Research (A*STAR), Singapore, Singapore

**Keywords:** Influenza A virus, H5N6, antibody-dependent cellular cytotoxicity, complement dependent cytotoxicity, antibody-dependent cellular phagocytosis, monoclonal antibody 9F4, vestigial esterase domain

## Abstract

The highly pathogenic avian influenza A (H5N6) virus has caused sporadic human infections with a high case fatality rate. Due to the continuous evolution of this virus subtype and its ability to transmit to humans, there is an urgent need to develop effective antiviral therapeutics. In this study, a murine monoclonal antibody 9F4 was shown to display broad binding affinity against H5Nx viruses. Furthermore, 9F4 can neutralize H5N6 pseudotyped particles and prevent entry into host cells. Additionally, ADCC/ADCP deficient L234A, L235A (LALA) and CDC deficient K322A mutants were generated and displayed comparable binding affinity and neutralizing activity as wild type 9F4 (9F4-WT). Notably, 9F4-WT, 9F4-LALA and 9F4-K322A exhibit *in vivo* protective efficacies against H5N6 infections in that they were able to reduce viral loads in mice. However, only 9F4-WT and 9F4-K322A but not 9F4-LALA were able to reduce viral pathogenesis in H5N6 challenged mice. Furthermore, depletion of phagocytic cells in mice lungs nullifies 9F4-WT's protection against H5N6 infections, suggesting a crucial role of the host's immune cells in 9F4 antiviral activity. Collectively, these findings reveal the importance of ADCC/ADCP function for 9F4-WT protection against HPAIV H5N6 and demonstrate the potential of 9F4 to confer protection against the reassortant H5-subtype HPAIVs.

## Introduction

Since its emergence in 1996, the highly pathogenic avian influenza virus (HPAIV) of H5N1 subtype (A/goose/Guangdong/1/96, or Gs/GD) has spread among poultry and wild birds, and sporadic human infections have been reported in Asia, Africa and Europe [1]. The haemagglutinin (HA) genes of the HPAIV H5N1 evolved into 10 phylogenetic clades (0–9), and clades 2 and 7 have further evolved into many subclades, but with no evidence of gene exchange between influenza viruses [2,3]. Since 2008, HPAI subtypes H5N2, H5N5, and H5N8 bearing the genetic backbone of the Gs/GD lineage H5 clade 2.3.4 have been identified, and these subtypes have subsequently evolved into different subclades including 2.3.4.4 [4–6]. The HA genes from clade 2.3.4.4 HPAIV H5N1 have undergone genetic reassortment with neuraminidase (NA) and various other genes of low pathogenic AIVs. As a result, newly emerging HPAIV of the H5N2, H5N3, H5N5, H5N6, H5N8 and H5N9 subtypes, collectively referred to as H5Nx viruses, have been spreading in poultry and wild birds in various countries of Asia, Europe and North America [7–12]. In April 2014, human infection with HPAIV H5N6 was first reported in the Sichuan province of China [13]. To date, the HPAI H5N6 viruses have also caused a total of 23 laboratory-confirmed cases of human infection, including seven deaths in China since 2014 (as of 7 June 2019; https://www.who.int/docs/default-source/wpro—documents/emergency/surveillance/avian-influenza/ai-20190607.pdf?sfvrsn=30d65594_24). Notably, it has been reported that H5N6 AIVs contain gene segments derived from a variety of AIV subtypes [14–19] and at least six genotypes arising from segment reassortment, including a rare variant that possesses the HA gene derived from H5N1 clade 2.3.2 [20]. Thus, there is still a risk that circulating HPAI H5N1 viruses will give rise to new dangerous reassortants or acquire the ability to transmit directly between humans, and this warrants the need for broadly neutralizing monoclonal antibodies (mAbs) against newly emerging H5 viruses.

The overall therapeutic effectiveness of a mAb against influenza infections is governed primarily by the binding epitopes of the mAb. While neutralizing antibodies against the various proteins of the influenza A virus (IAV) have been reported, HA targeting antibodies have been the foci of many studies because they have been known to protect against IAV infections [21,22]. HA targeting antibodies are further classified into head or stalk binding antibodies. Neutralizing antibodies that bind to the head region of HA are generally able to directly inhibit virus entry without additional Fc mediated responses such as antibody-dependent cellular cytotoxicity (ADCC), complement dependent cytotoxicity (CDC) or antibody-dependent cellular phagocytosis (ADCP) but are often only effective against specific strains of IAV [23]. In contrast, stalk-binding antibodies are less potent at direct neutralization of IAV and rely on Fc mediated responses for virus neutralization, but are able to inhibit different subtypes of IAV [23–25]. These effector functions can act against both free virions and virus-infected cells [26]. Fc-receptor function, particularly ADCC, is vital to the utility of stalk-specific antibodies. Substitutions of two leucine (L) residues to alanine (A) at position 234 and 235 (e.g. LALA) were demonstrated to reduce Fc binding to Fc gamma receptors (FcγRs) [27] to consequently decrease ADCC, ADCP and reduce complement binding/activation [28–32]. An earlier study showed that mice administered a FcR-binding deficient mutant (FI6-LALA) of the stalk-specific neutralizing mAb FI6, were less likely to survive a lethal dose of influenza virus compared to the wild type mAb FI6 [33].

Besides head or stalk binding antibodies, several neutralizing mAbs binding to the vestigial esterase (VE) domain of HA have been reported recently [34–38]. The murine mAb 9F4 was isolated from a mouse immunised with recombinant HA protein from the A/chicken/Hatay/2004 (H5N1) strain. 9F4 binds to the VE region of HA, does not affect IAV viral attachment but instead prevents viral entry by inhibiting post-endocytosis membrane fusion of IAV to the endosome. Furthermore, 9F4 was demonstrated to bind to different clades of H5N1 IAV and was able to confer protection in mice challenged with A/smew/Sweden/V820/06 (H5N1) [38]. These observations are consistent with other reported mAbs that bind unique epitopes of the VE domain of various types of influenza viruses. Collectively, the information currently available suggests that VE-binding antibodies generally inhibit virus entry by blocking membrane fusion and are able to neutralize different virus clades within the same virus subtype [39]. All three reported mAbs 100F4 [40], 46B8 [37] and H3v-47 [34] were also capable of initiating ADCC, although ADCC was only shown to be essential for 100F4 but was not yet assessed in 46B8 and H3v-47.

In this study, we assessed the binding affinity of the VE targeting antibody 9F4 against several H5Nx viruses and demonstrate that binding to H5Nx is consistent with conservation of their binding epitopes. Furthermore, we also establish that therapeutic utility of 9F4 against H5N6 infections *in vivo* likely depend on the host’s alveolar macrophages for antiviral protection. Our observation is similar with previous studies detailing the importance of alveolar macrophages for protection against influenza infections by non-neutralizing broadly reactive antibodies in a monoclonal [41,42] or polyclonal setting [42]. We focused on HPAI H5N6 viruses as these have been associated with human cases of infection as well as their increasing prevalence in poultry and wild birds worldwide. Furthermore, it has been reported that H5N6 virus is gradually becoming more prevalent in poultry than H5N1 virus in China [43]. Comparisons between 9F4-WT, an ADCC and ADCP deficient mutant 9F4-LALA and a CDC deficient 9F4-K322A against a mouse IgG2a isotype control revealed that the ADCP and/or ADCC but not the CDC pathways contributes significantly to the protective role of 9F4. Furthermore, 9F4-WT showed higher antiviral potency than 9F4-LALA in that 9F4-WT treated mice had better survival rates and displayed less severe histopathological changes. To our knowledge this is the first study investigating the importance of CDC and involvement of alveolar macrophages for the antiviral function by a VE targeting mAb. Finally, consistent with a previous study investigating a stalk-binding antibody [44], 9F4-WT was also protective against H5N6 infection when administered via the intranasal route.

## Materials and methods

### Cells and viruses

Madin-Darby canine kidney (MDCK) cells and African green monkey kidney fibroblast (COS-7) cells were obtained from the American Type Culture Collection and grown in Dulbecco’s Modified Eagle’s Medium (DMEM; HyClone) supplemented with 10% foetal bovine serum (FBS; HyClone), and penicillin/streptomycin (Thermo Fisher Scientific). 293FT cells were purchased from Invitrogen and grown in DMEM containing 2 mM glutamine (Thermo Fisher Scientific), 0.1 mM nonessential amino acids (Thermo Fisher Scientific), and 500 µg/ml geneticin (Thermo Fisher Scientific). 293 suspension cells were cultured in Freestyle F17 expression media (Thermo Fisher Scientific) supplemented with 0.1% Pluronic® F-68 (Thermo Fisher Scientific), 4 mM L-glutamine, and 25 µg/ml geneticin. The recombinant influenza virus H5N6 was generated by eight-plasmid-based reverse genetics containing seven segments from A/Puerto Rico/1934 and the HA segment of A/Guangzhou/39715/2014 as previously described [45]. The HA gene was obtained via gene synthesis (Bio Basic). All virus work pertaining to the generation, propagation, detection of rgPR8 H5N6 and animal experimentation was carried out in a BSL3+ or ABSL3 facility (National University of Singapore).

### Production and purification of monoclonal antibodies

The VH and VL genes of 9F4 were cloned into pFUSEss-CHIg-mG2a and pFUSE2ss-CLIg-mK cloning vectors (InvivoGen) respectively in order to generate mouse IgG2a wild type 9F4. Amino acid substitution K322A in the fragment crystallisable region (Fc region) of 9F4-pFUSEss-CHIg-mG2a was introduced by site-directed mutagenesis. Briefly, 293 suspension cells cultured in baffled flasks were diluted to 1.0 × 10^6^ cells/ml and co-transfected with 0.6 μg/ml of pFUSEss-CHIg-mG2a cloning vector containing VH of 9F4-WT or -K322A and 0.9 μg/ml of pFUSE2ss-CLIg-mK cloning vector containing VL gene of 9F4. pTT5 cloning vectors containing VH and VL of 9F4-LALA were also co-transfected as above. Antibodies expressed were purified using a HiTrap protein A affinity column. The column was eluted into fractions using 0.1 M glycine-HCl elution buffer (pH 2.7), and neutralized with sodium hydroxide. Fraction samples were analyzed using 10% sodium dodecyl sulphate polyacrylamide gel electrophoresis (SDS-PAGE) followed by Coomassie brilliant blue staining. Fraction samples were pooled and dialysed in phosphate buffered saline (PBS) overnight at 4°C. Dialysed samples were filter sterilized using Ultrafree-CL centrifugal filters (Millipore) and quantified with Coomassie plus assay reagent (BioRad).

### Enzyme-linked immunosorbent assay (ELISA)

96-well ELISA plates (Nunc™ Maxisorp™) were coated overnight at 4°C with 100 ng of purified haemagglutinin (HA) proteins of H5Nx [A/Vietnam/1194/2004(H5N1); A/chicken/Iowa/04-20/2015(H5N2); A/duck/Guangdong/GD01/2014(H5N6); A/broiler duck/Korea/Buan2/2014(H5N8)], A/Missouri/09/2014(H3N2), A/Netherlands/219/2003(H7N7), A/Anhui/DEWH72-01/2013(H7N9), A/guinea fowl/Hong Kong/WF10/99(H9N2) purchased from Sino Biological, washed with PBS containing 0.05% Tween-20 (PBST) and blocked with 5% FBS/PBST for 1 h. Serially diluted mAbs in 5% FBS/PBST were added to the plates and incubated for 1.5 h at 37°C. A mouse IgG2a mAb, 1A4, which was generated using the hepatitis C virus NS5B protein, was also used at the highest concentration (50 ng/ml) as an isotype control antibody [46]. The plates were washed and incubated with horseradish-peroxidase-conjugated goat anti-mouse IgG antibody (Thermo Fisher Scientific) for 1 h at 37°C. The plates were washed three times with PBST before the reaction was visualized using the substrate 3,3′,5,5′-tetramethylbenzidine (TMB; Thermo Fisher Scientific) and halted by the addition of sulphuric acid. The absorbance at 450 nm was measured using a plate reader (Tecan Infinite M200) and normalized against the background of bovine serum albumin-coated wells.

### Pseudotyped lentiviral particle neutralization assay

Lentiviral pseudotyped particles harbouring the HA glycoprotein (HApp) were generated by co-transfection of 293FT cells with HA expressing plasmids and the envelope-defective pNL4.3.Luc.R^−^E^−^ proviral genome. The HA expressing plasmids were constructed as follows: HA genes [A/Vietnam/1203/2004(H5N1); A/chicken/Iowa/04-20/2015(H5N2); A/Guangzhou/39715/2014(H5N6); A/broiler duck/Korea/Buan2/2014(H5N8)] were obtained via gene synthesis (Bio Basic). The respective HA genes were then cloned into the pXJ3’ expression vector. All sequences were confirmed by DNA sequencing (Bio Basic). Similar to other studies, the NA gene from A/chicken/Hatay/2004 (H5N1; GenBank accession number AJ867075) was also co-transfected to facilitate the release of pseudotyped particles from the 293FT producer cells [47–50]. The culture supernatants were collected 48 h post-transfection (hpt), filtered, and stored at −80°C until use. HApp titre was determined using a lentivirus-associated p24 ELISA kit (Cell Biolabs). MDCK cells were seeded in 96-well plates 24 h before infection. Briefly, mAbs were 10-fold serially diluted as indicated and mixed with an equal volume of pseudotyped particles for 1 h at room temperature (RT) before the mixture was added to target cells for infection. A control mAb 1A4 of the same isotype (IgG2a) was also used at the highest concentration (10 μg/ml). Cells were incubated at 37°C for 48 h and were equilibrated to ambient temperature prior to the addition of Bright-Glo^TM^ reagent (Promega) for 5 min. Luminescence was measured in triplicates using a plate reader, and the data were expressed as a percentage of the reading obtained in the absence of antibody (No Ab), which was set at 100%. Three independent experiments were performed and the average readings are presented.

### C1q binding assay

96-well ELISA plates (Nunc™ Maxisorp™) were coated overnight at 4°C with 100 ng of purified HA protein of H5N6 (A/duck/Guangdong/GD01/2014), washed with PBS containing 0.05% Tween-20 (PBST) and blocked with 5% bovine serum albumin (BSA)/PBS for 1 h. Serially diluted mAbs in 0.1% BSA/PBST were added to the plates and incubated for 1.5 h at RT. 1A4 was used at the highest concentration (1 µg/ml) as an isotype control antibody. 2 µg/ml human C1q protein (Abcam) was then added and incubated for 2 h at RT, followed by the incubation for 1 h at RT with mouse anti-huC1q (1:200 dilution) conjugated to biotin (Abcam). Then, neutravidin-HRP (1:10,000 dilution) was added for 1 h at RT before the reaction was visualized using TMB and halted by the addition of sulphuric acid. The absorbance at 450 nm was measured using a plate reader. To correct for background binding, absorbance of wells without C1q addition were subtracted.

### Transient transfection and expression of H5N6-HA protein

COS-7 cells were seeded into a sterile 6-cm dish and transfected using Lipofectamine™ LTX reagent with PLUS™ reagent (Thermo Fisher Scientific) according to the manufacturer’s protocol. Briefly, 5.5 μg plasmid DNA of A/Guangzhou/39715/2014(H5N6) was diluted into 1 ml of Opti-MEM® I medium (Thermo Fisher Scientific). 5.5 μl of PLUS™ reagent was added directly to the diluted DNA and incubated for 10 min at RT. 16.5 μl of Lipofectamine® LTX was then added into the mixture and incubated for 25 min at RT. Subsequently, 1 ml of the DNA-Lipofectamine® LTX complexes was added dropwise to the dish containing a monolayer of cells in complete growth medium. Cells were incubated for 6 h at 37°C and then detached by trypsinisation. The cells were resuspended in 10% FBS-DMEM, seeded in transparent or white 96-well plates at a density of 1.5 × 10^4^ cells per well and incubated overnight at 37°C. Cells expressing HA protein of H5N6 on the cell surface were used for the murine FcγRIV (mFcγRIV) ADCC reporter bioassay.

### mFcγRIV ADCC reporter bioassay

ADCC reporter bioassay (Promega) was performed according to the manufacturer’s instructions, with modifications for use with adherent target cells. COS-7 cells were transfected as above and seeded in white 96-well plates. 9F4-WT, 9F4-LALA, 9F4-K322A and 1A4 were prepared at 5-fold serial dilutions, starting at a concentration of 5 µg/ml and added to the plated target cells for 45 min at RT. Murine FcγRIV effector cells were rapidly thawed and diluted in ADCC assay buffer before addition to the cells expressing surface H5N6-HA and incubated for 6 h at 37°C in a humidified 5% CO_2_ incubator. After incubation, the cells were equilibrated to ambient temperature and incubated with Bio-Glo^TM^ reagent for 5 min. Luminescence was measured in triplicates using a plate reader, and the data were expressed as fold induction above background (0 μg/ml antibody control). Three independent experiments were performed and the average readings are presented.

### Ethics statement

Animal experiments were performed according to the approved institutional animal care and use committee (IACUC) protocol pertaining to the development of H5N1 neutralizing antibodies *in vivo* (R14-1025) at the National University of Singapore.

### Mouse husbandry, infection and administration of liposomal clodronate

Female BALB/c mice were purchased from the Jackson Laboratory and housed under ABSL3 conditions in the ABSL3 facility at the National University of Singapore. Seven to nine weeks old mice were used for infection experiments. Mice were weighted and anaesthetized via intraperitoneal injections of ketamine (75 mg/kg) and medetomidine (1 mg/kg) formulations (Comparative Medicine, National University of Singapore) prior to infections or intranasal instillation of liposomal clodronate (Encapsula NanoSciences LLC). To infect mice with 100 PFU of rgPR8 H5N6, virus stocks were diluted in PBS to a concentration of 5 PFU/µl before a 20 µl volume was administered into sedated mice via intranasal instillations. Mock-infected mice were given a 20 µl volume of PBS intranasally instead. To reverse the effects of anaesthesia, infected mice were then intraperitoneally given a 1 mg/kg dose of atipamezole (Comparative Medicine, National University of Singapore). To deplete alveolar macrophages, mice were given two 20 µl doses of liposomal clodronate or control liposomes, one dose 2 days prior to infection and a second dose simultaneously during infection. Mice were monitored daily for their health status and body weights over a period of 14 days; mice that lost more than 25% of their initial body weight were marked as non-survivors and euthanised. Mice euthanasia was performed via carbon dioxide asphyxiation according to IACUC guidelines.

### Therapeutic administration of 9F4

To assess the protective capabilities of 9F4-WT, 9F4-LALA and 9F4-K322A against H5N6 infections, rgPR8 H5N6 infected mice were given a single 10 mg/kg dose of 9F4-WT, 9F4-LALA, 9F4-K322A or an irrelevant isotype control 1A4 as a therapeutic via 200 µl intraperitoneal injections 24 h post-infection (hpi). For intranasal therapy, mice were sedated as described in the previous section before receiving a single dose of antibody at 2 mg/kg intranasally in a final volume of 20 µl, 24 hpi. Intranasally treated mice were then given atipamezole intraperitoneally to reverse the effects of anaesthesia. Mock-infected mice were given equivalent volumes of PBS intraperitoneally or intranasally during the therapeutic phase.

### Histology and viral plaque assay

Mice lungs were collected at 4 or 14 days post-infection (dpi), halved and fixed in 10% neutral buffered formalin (Sigma Aldrich) for at least 16 h before they were dehydrated, embedded in paraffin and prepared into 5 micron sections. Lung sections were stained with Haematoxylin and Eosin (H&E), and examined for the presence of histopathological abnormalities.

The remaining lung halves collected 4 dpi were immersed into 1 ml of PBS, homogenized in whirl-pak sampling bags (Sigma Aldrich), transferred into microfuge tubes and centrifuged at 6000x g for 10 min at 4°C. Supernatant fractions were then transferred into fresh tubes and stored at −80°C. To perform virus plaque assays, supernatants were thawed, serially diluted with PBS and applied onto MDCK cells at 90% confluency for 1 h at 37°C to allow virus adsorption. Cells were subsequently rinsed with pre-warmed PBS and cultured in MEM (Thermo Fisher Scientific) supplemented with 1.2% Avicel® (FMC Biopolymer) and 2.5 µg/ml trypsin (Thermo Fisher Scientific). After 2 days of incubation, cells were fixed with 10% neutral buffered formalin for at least 16 h before plaques were visualized and quantified by staining with 0.1% crystal violet solution (Sigma Aldrich).

### Histopathological analyses

H&E stained lung sections were evaluated as a blinded study by a board-certified pathologist at the Advanced Molecular Pathology Laboratory (AMPL) located in the Institute of Molecular and Cell Biology in Singapore. Key observations noted were presence of lung haemorrhage, degeneration and necrosis of the bronchial and alveolar epithelium, suppurative inflammation defined by the presence of polymorphonuclear leukocytes and/or mononuclear cell inflammation characterized by presence of lymphocytes, plasma cells and macrophages.

### Purification and flow cytometry analysis of mononuclear cells from mice lungs

After 6 days post liposome administration, mice lungs were minced with sterile scalpels in RPMI (Hyclone) supplemented with 10% FBS and incubated in RPMI, 10% FBS, 0.8 mg/ml type IV collagenase (Thermo Fisher Scientific), 1 µg/ml DNase I (Sigma Aldrich) at 37°C for 1 h before they were homogenized with a 70 µm strainer (BD Falcon). Cells were washed once with RPMI, overlaid onto a 40/70% percoll gradient (GE Healthcare) and centrifuged at 800x g for 20 min without brakes. Mononuclear cell fractions were collected, subjected to ACK lysis (Thermo Fisher Scientific) for 5 min on ice to remove erythrocytes and stained with antibodies against CD45.2 (BD, 561874), Mac1 (BD, 553312), CD11c (BD, 558079) and Siglec F (BD, 552126), washed and counterstained with DAPI before analysis with a BD LSRII flow cytometer. Cell populations were defined as eosinophils (CD45.2^+^ Mac1^+^ Siglec F^+^), interstitial macrophages (IM: CD45.2^+^ Mac1^+^ Siglec F^−^ CD11c^±^) and alveolar macrophages (AM: CD45.2^+^ Mac1^−^, Siglec F^+^ CD11c^+^) [41,51].

### Statistical analysis

Statistical significance was determined using unpaired, two-tailed Student’s t-tests with Welch’s correction for unequal variances for virus plaque assays (Figures 4–7C) or a two-way ANOVA and Tukey’s multiple comparisons tests for *in vitro* ADCC and CDC assays (Figure 3; GraphPad Prism). *P* values <0.05 were considered statistically significant.

## Results

### 9F4 displays broad binding affinity and neutralizing activity against H5Nx viruses

**Figure 1.**
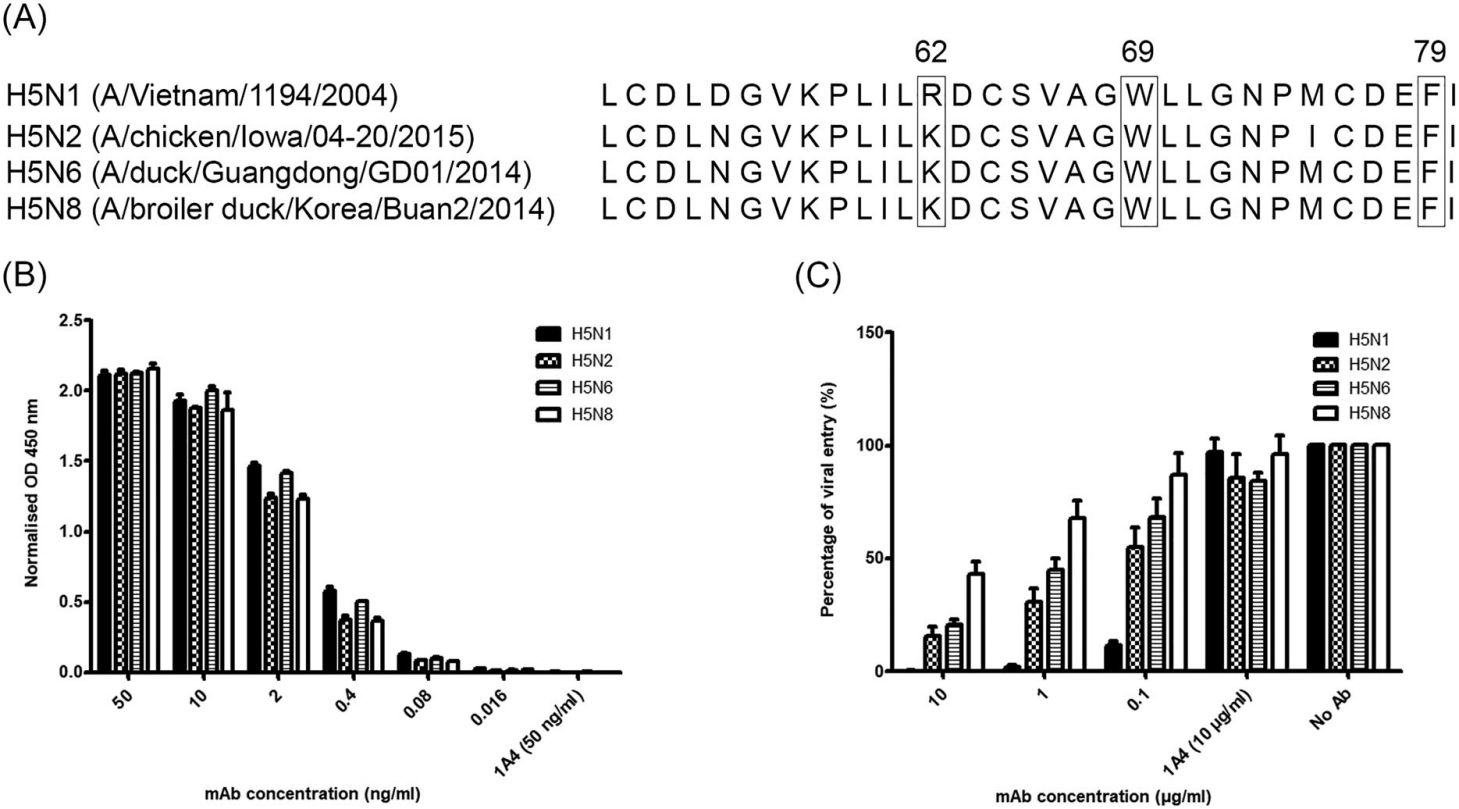
9F4 displays broad binding affinity and neutralizing activity against H5Nx viruses. (A) Alignment of residues 50–80 in the HA protein of H5Nx viruses with the corresponding domain in one clade 1 (VN04) and three other clade 2.3.4.4 viruses (Iowa15, GD14 and Korea14). For comparison, H3 numbering is followed. The conservation of R62, W69 and F79, which are important for the interaction with 9F4, are boxed. (B) ELISA was performed to determine the ability of 9F4 to bind to H5Nx-HA. Wells were coated with HA proteins of H5Nx (H5N1 (A/Vietnam/1194/2004); H5N2 (A/chicken/Iowa/04-20/2015); H5N6 (A/duck/Guangdong/GD01/2014); H5N8 (A/broiler duck/Korea/Buan2/2014)) and incubated with serially diluted 9F4. 1A4 was also used at the highest concentration (50 ng/ml) as an isotype control antibody. (C) Pseudotyped lentiviral particles harbouring the HA proteins of H5Nx IAV were incubated with 10-fold serially diluted 9F4-WT for 1 h at RT before inoculation onto MDCK cells. Luciferase activity in the cell lysates was determined at 48 hpi. Viral entry, as indicated by the luciferase activity, was expressed as a percentage of the reading obtained in the absence of antibody (No Ab), which was set at 100%. 1A4 was also used at the highest concentration (10 μg/ml) as an isotype control antibody. Representative data from three independent experiments are shown and error bars represent standard error of the mean (SEM) of the experiment carried out in triplicates.

As described previously, 9F4 binds to the VE domain of H5N1 HA and was shown to have cross clade neutralizing efficacy against clade 1 and up to fifth order subclade of clade 2 H5N1 virus [38,45,52]. Since 2014, novel reassortants of clade 2.3.4.4 HPAI H5Nx viruses have emerged and spread rapidly. In order to determine if 9F4 also binds HA of different subtypes of H5Nx viruses, ELISA was performed by using purified HA proteins of H5N1 (A/Vietnam/1194/2004), H5N2 (A/chicken/Iowa/04-20/2015), H5N6 (A/duck/Guangdong/GD01/2014) and H5N8 (A/broiler duck/Korea/Buan2/2014). Sequence alignment shows that there is a R62 to K62 substitution in the HA of H5N2, H5N6 and H5N8 whereas W69 and F79 are completely conserved (Figure 1A). Nevertheless, the ELISA readings were similar for all these HA proteins and 9F4 bound to H5Nx HA in a dose dependent manner (Figure 1B). No significant binding was observed when an irrelevant mAb 1A4 was used at the highest concentration of 50 ng/ml. These results indicate that 9F4 displays broad binding affinity against H5Nx viruses, and the R62 to K62 substitution did not significantly affect the binding of 9F4. This may be expected given that arginine and lysine have similar chemical characteristics. Moreover, we investigated further using other subtypes and performed similar alignment of residues 50–80 in the HA protein of H5N1, H3N2, H7N7, H7N9, H9N2 viruses (Supplementary Figure S1A). As expected, 9F4 did not bind to other subtypes (Supplementary Figure S1B), suggesting that a combination of the three non-contiguous residues is crucial for binding and at least two of the residues must be conserved to allow binding to 9F4.

To further analyse the neutralizing activity of 9F4 against H5Nx viruses, we produced lentiviral pseudotyped particles harbouring the HA glycoprotein of H5Nx viruses. Figure 1C shows that 9F4 neutralized the pseudoviruses to different extents with the strongest neutralization elicited against H5N1. In general, there was a dose dependent neutralizing activity observed in all H5Nx pseudoviruses. On the other hand, irrelevant mAb 1A4 did not show any significant neutralizing activity. In previous studies [38,45,52], we have only shown that 9F4 binds and neutralizes up to H5 clade 2.3.2.1a, thus this study shows for the first time that 9F4 also binds and neutralizes the H5 clade 2.3.4.4 which may have high reassortant capability.

### 9F4-WT and its Fc mutants display similar binding affinities to H5N6-HA protein and neutralize H5N6 pseudotyped particles

**Figure 2.**
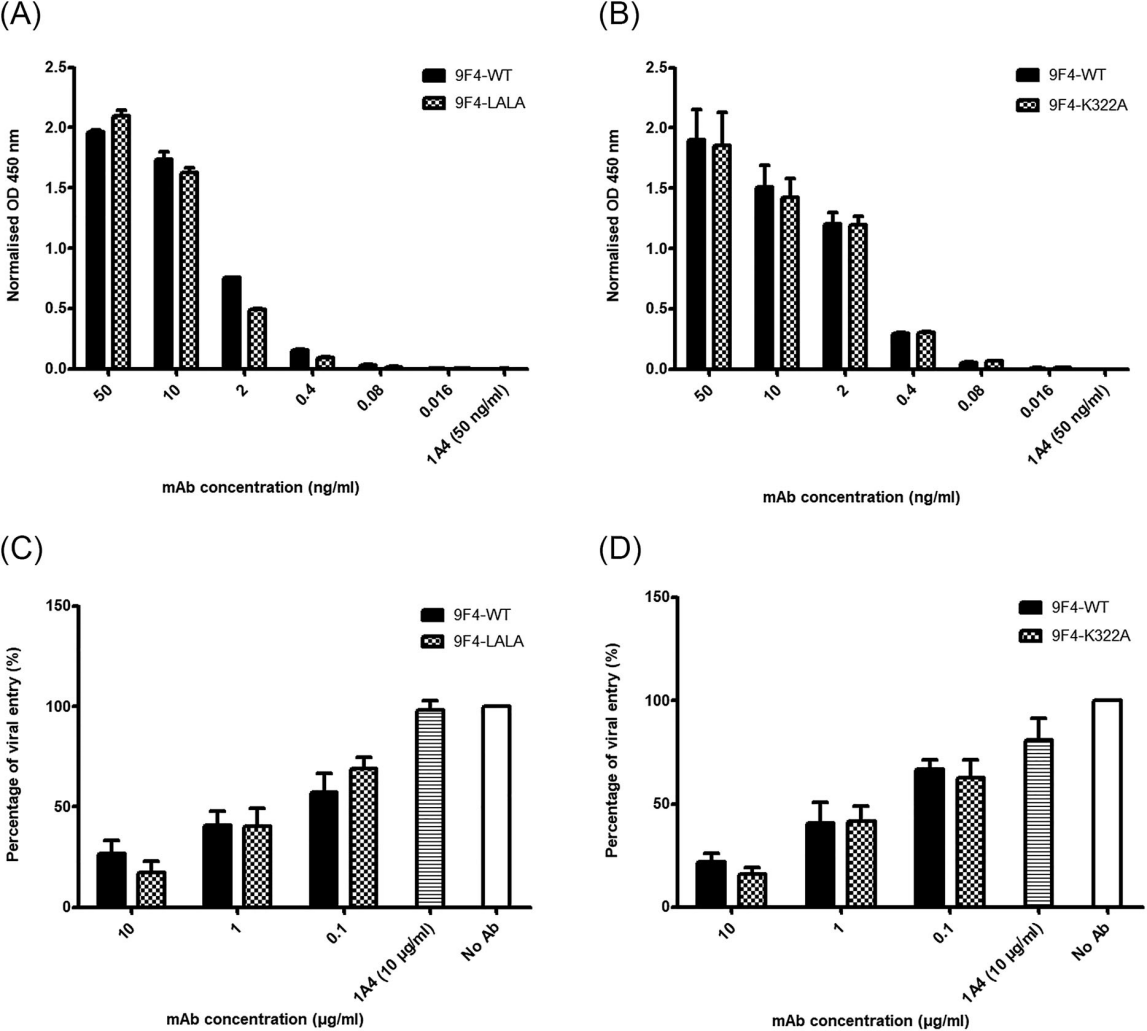
9F4-WT and its Fc mutants display similar binding affinities to H5N6-HA protein and neutralize against H5N6 pseudotyped particles. ELISA was performed to compare the binding affinity of 9F4-WT with (A) 9F4-LALA or (B) 9F4-K322A to H5N6-HA. Wells were coated with H5N6-HA protein and incubated with serially diluted mAbs as indicated. 1A4 was also used at the highest concentration (50 ng/ml) as an isotype control antibody. Pseudotyped lentiviral particles harbouring the HA proteins of H5N6 IAV were incubated with 10-fold serially diluted 9F4-WT, (C) 9F4-LALA or (D) 9F4-K322A for 1 h at RT before inoculation onto MDCK cells. Luciferase activity was determined as above. Representative data from three independent experiments are shown and error bars represent SEM of the experiment carried out in triplicates.

Since the highly pathogenic H5N6 strain poses a global health concern, our work focuses on investigating the antiviral function of 9F4 against H5N6 infections. Furthermore, some mAbs are able to trigger a variety of effector mechanisms, such as Fc-mediated recruitment of effector cells for ADCC or ADCP via FcγR activation, and CDC. In order to investigate whether these effector mechanisms play a role in the antiviral function of 9F4 against H5N6 infections, we have constructed two Fc mutants, namely 9F4-LALA and 9F4-K322A, by introducing point mutations L234A, L235A [28,30] and K322A [28,53], respectively in the Fc region of 9F4. To demonstrate that the introduction of the Fc mutations did not influence the affinity of the antibody for HA protein, we performed binding studies to H5N6-HA by ELISA (Figure 2A and B). As expected, the 9F4-WT and its Fc mutants bound with similar affinities to H5N6-HA.

Furthermore, we also tested the three mAbs in a neutralization assay with lentiviral pseudotyped particles harbouring the H5N6-HA glycoprotein. All three mAbs neutralized against H5N6 pseudotyped particles similarly in a dose dependent manner and prevented entry into host cells (Figure 2C and D). In contrast, irrelevant mAb 1A4 did not show any neutralizing activity.

### mFcγRIV activation is induced by 9F4-WT and 9F4-K322A but not 9F4-LALA

**Figure 3.**
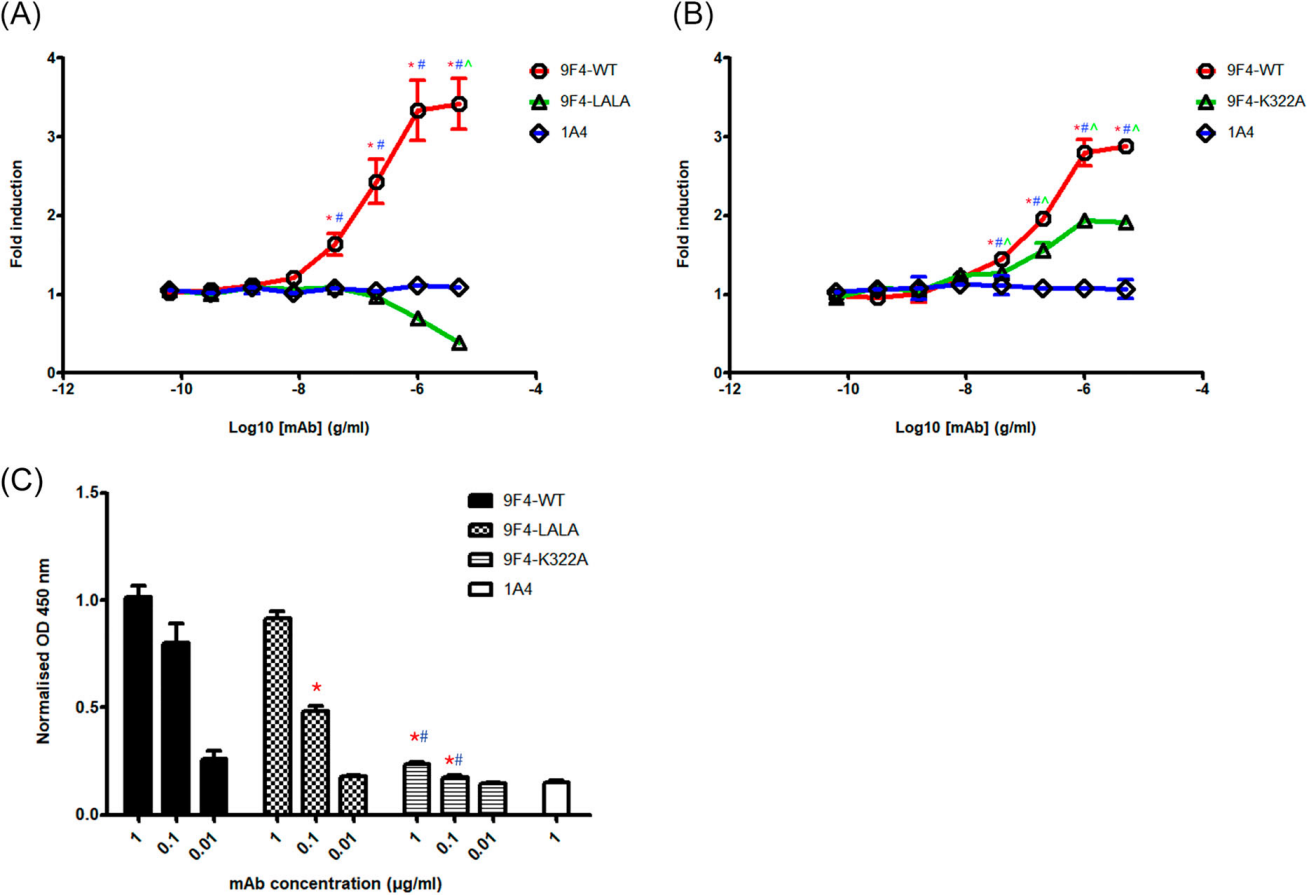
mFcγRIV activation is induced by 9F4-WT and 9F4-K322A but not 9F4-LALA. Mouse FcγRIV-based ADCC reporter assay was performed for 9F4-WT, (A) 9F4-LALA and (B) 9F4-K322A as described in materials and methods. 1A4 was used as an isotype control. Representative data of three independent experiments carried out in triplicates are shown and error bars represent SEM between replicates. Symbols represent statistically significant differences at *p* < 0.05 when comparing 9F4-WT with its Fc mutants (red asterisk) or 1A4 (blue hash), or Fc mutants with 1A4 (green carat). (C) Binding of human C1q to 9F4-K322A is abolished. C1q binding ELISA was performed to compare the binding affinity of human recombinant C1q to 9F4-WT and its Fc mutants. Wells were coated with H5N6-HA protein and incubated with serially diluted mAbs, followed by the addition of c1q protein. 1A4 was also used at the highest concentration (1 μg/ml) as an isotype control antibody. Representative data from three independent experiments are shown and error bars represent SEM of the experiment carried out in triplicates. Symbols represent statistically significant difference at *p* < 0.05 when compared to 9F4-WT (red asterisk) or 9F4-LALA (blue hash).

Since we have demonstrated that 9F4-WT and its Fc mutants display similar binding and neutralizing activity against H5N6, we next determined their ability to mediate ADCC using an *in vitro* bioassay. With the addition of mFcγRIV effector cells wherein all components are co-cultured, the antibody will simultaneously bind the target cell antigen and mFcγRIV receptors on the effector cell surface, resulting in NFAT-RE-mediated luciferase activity. As shown in Figure 3A, 9F4-WT was able to bind and activate mFcγRIV with a 3-fold induction, whereas 9F4-LALA exhibited virtually no mFcγRIV activation, even at the highest concentration of 5 µg/ml. Similarly, the fold induction of 9F4-WT was significantly higher as compared to that of 1A4. On the other hand, 9F4-K322A showed induction of mFcγRIV activation but the efficacy in eliciting ADCC was 1-fold lower than 9F4-WT (Figure 3B). Nevertheless, the fold induction of both 9F4-WT and 9F4-K322A were significantly higher as compared to that of 1A4. These results suggest that 9F4-WT and 9F4-K322A possess Fc binding affinity for mFcγRIV and are able to induce mFcγRIV signalling for effecting ADCC or ADCP.

### K322a mutation significantly reduces 9F4’s ability to bind C1q

Besides ADCC or ADCP, antibodies can also induce complement activation. Activation of the classical complement pathway results from binding of C1q to the Fc domain of antibodies bound to virus-infected cells [54]. To determine if 9F4-K322A can bind C1q, we performed a C1q binding ELISA. Indeed, we observed reduced binding of C1q by 9F4-K322A compared to that of 9F4-WT or 9F4-LALA (*p* < 0.0001; Figure 3C). Reduced binding of C1q by 9F4-LALA compared to 9F4-WT (*p* < 0.0001) was detected at a lower antibody concentration of 0.1 µg/ml although similar levels of binding by both antibodies were observed when 1 µg/ml of antibody were present (*p* > 0.05). 1A4 was included as an isotype control; no binding of C1q by 1A4 was detected, presumably because 1A4 did not bind to H5N6-HA.

### 9F4-WT and 9F4-LALA confer *in vivo* protection against H5N6 infection

The ability of 9F4-WT to confer protection against H5N1 (A/smew/Sweden/V820/06) infections was previously shown [38]. To assess if 9F4 would also be able to protect against the emerging H5N6 strain, mice were intranasally infected with 100 PFU of rgPR8 H5N6 recombinant virus. Infected mice received a single therapeutic dose of 9F4-WT, 9F4-LALA, or an irrelevant isotype control 1A4 at 10 mg/kg, 24 hpi via the intraperitoneal route.

**Figure 4.**
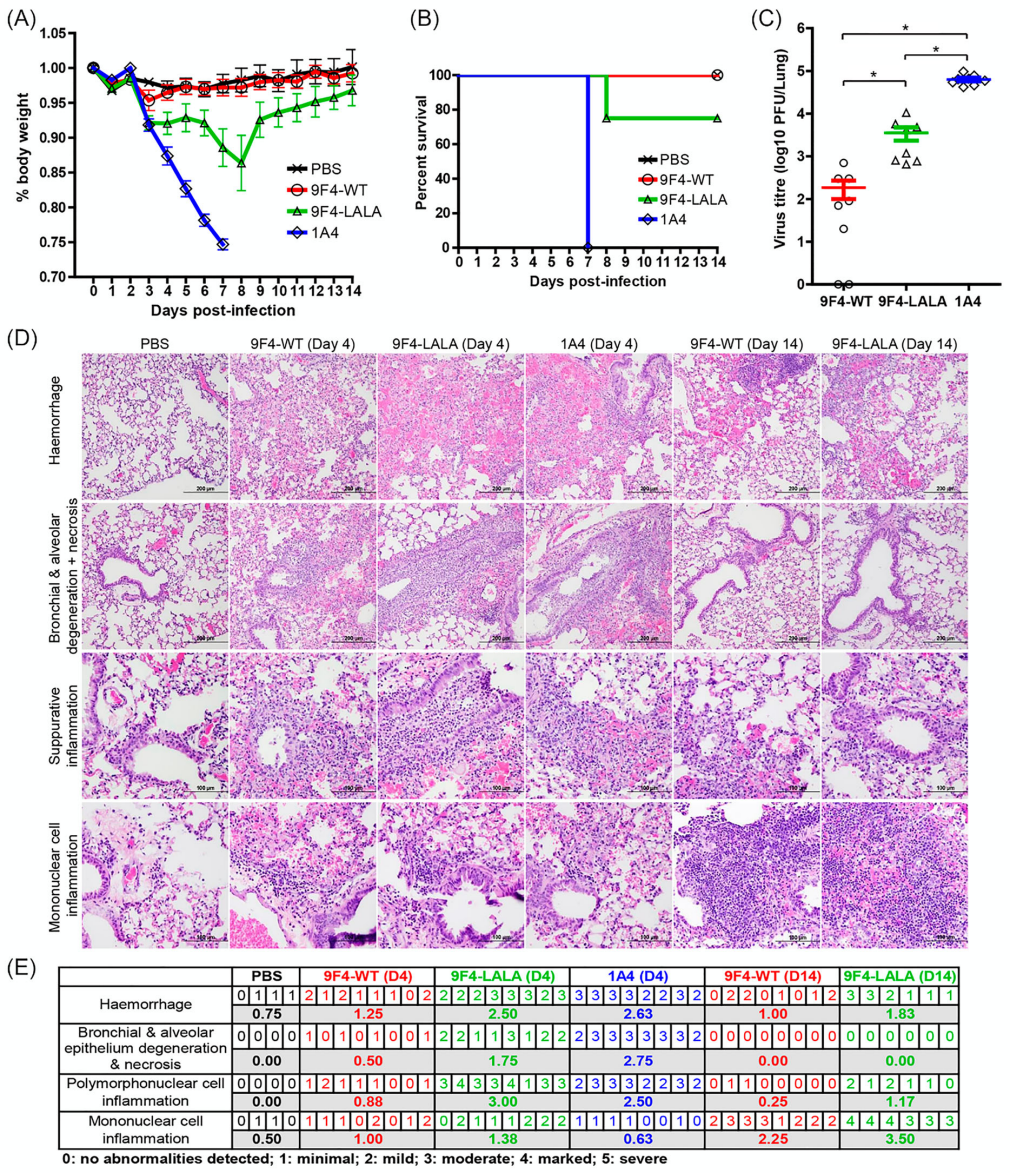
ADCC and/or ADCP contributes to the *in vivo* protective function of 9F4. Balb/c mice were infected with 100 PFU of rgPR8 H5N6 and received a single therapeutic dose of 9F4-WT, 9F4-LALA or the isotype control 1A4 at 10 mg/kg via intraperitoneal injections 24 hpi. (A) Body weight profiles and (B) animal survival are shown. (C) Lung virus titres were determined at 4 dpi by plaque assays with MDCK cells. *n* = 8 per treatment group, error bars represent SEM, asterisks indicate *p* < 0.05. (D) Representative images of H&E stained formalin fixed paraffin embedded lung sections along with their (E) corresponding histopathological grades of individual mice and averages of treatment groups at 4 or 14 dpi are shown.

As expected, all of the infected mice that received 1A4 began losing weight steadily 3 dpi and died at 7 dpi. Infected mice treated with 9F4-WT showed modest weight loss of approximately 5% body weight at day 3 but regained normal body weight profiles similar to that of mock-infected mice by 5 dpi (Figure 4A). All 9F4-WT treated mice survived H5N6 infections (Figure 4B). In 9F4-LALA treated mice, weight loss was also observed between 3 and 8 dpi although the degree was less severe as compared to 1A4 treated mice. 25% (2 of 8 mice) of 9F4-LALA treated mice died at 8 dpi whereas the remaining mice survived and started to regain body weight between 9 and 14 dpi (Figure 4A and B).

**Figure 5.**
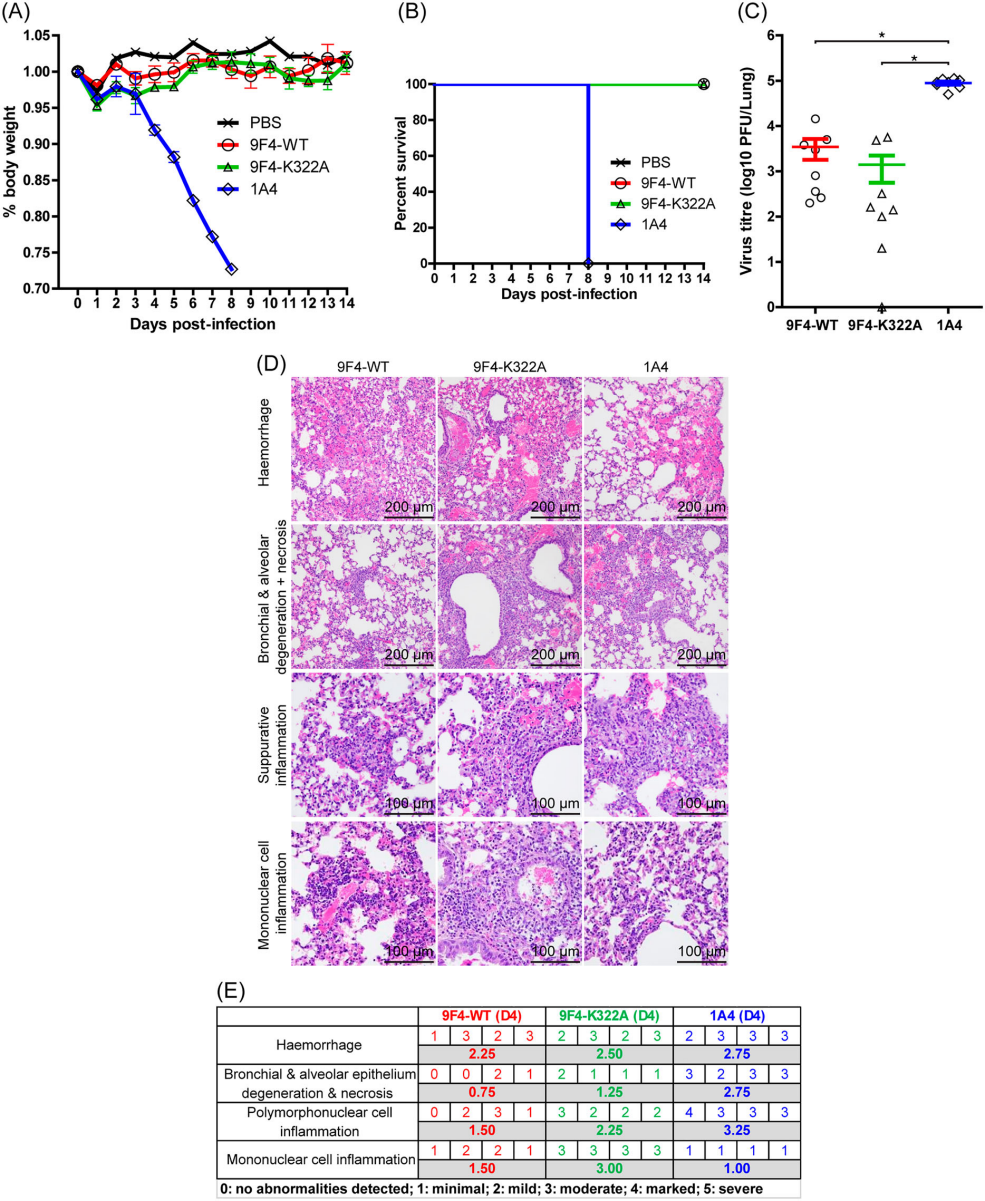
CDC is dispensable for the *in vivo* protective function of 9F4. Balb/c mice were infected with 100 PFU of rgPR8 H5N6 and received a single therapeutic dose of 9F4-WT, 9F4-K322A or the isotype control 1A4 at 10 mg/kg via intraperitoneal injections 24 hpi. (A) Body weight profiles and (B) animal survival are shown. *n* = 4. (C) Lung virus titres were determined at 4 dpi by plaque assays with MDCK cells. *n* = 8 per treatment group. Error bars represent SEM, asterisks indicate *p* < 0.05. (D) Representative images of H&E stained formalin fixed paraffin embedded lung sections along with their (E) corresponding histopathological grades of individual mice and averages of treatment groups at 4 dpi. *n* = 4 per treatment group.

Analysis of viral titres present in mice lungs at 4 dpi with H5N6 revealed a significant reduction of virus in 9F4-WT (1.48 × 10^2^ PFU/lung; ∼427-fold reduction) and 9F4-LALA (3.56 × 10^3^ PFU/lung; ∼17-fold reduction) treated mice compared to mice that received an irrelevant isotype control (6.32 × 10^4^ PFU/lung). A comparison between 9F4-WT and 9F4-LALA also showed significantly lower virus titres in the 9F4-WT treated group (Figure 4C). Collectively, the ability of 9F4-WT and 9F4-LALA treatments to confer survival and reduce IAV titres in the lungs of H5N6 infected mice demonstrate their antiviral efficacy against H5N6 IAVs.

### 9F4-WT but not 9F4-LALA treatment is able to reduce virus pathogenicity at 4 dpi

When analyzed at 4 dpi, histopathological analyses of the lungs of mice infected with H5N6 and treated with the irrelevant antibody 1A4, revealed extensive tissue damage in the form of haemorrhage as well as degeneration and necrosis of the bronchial and alveolar epithelium (Figure 4D and E). The presence of suppurative and mononuclear cell inflammations indicative of the acute phase and chronic phases of the inflammatory response respectively were also noted. This is consistent with the high viral load in the lung and rapid weight loss observed in this group of mice and reflects a lack of protection against H5N6 infection. In contrast, minimal levels of lung haemorrhage possibly due to carbon dioxide asphyxiation during euthanasia and mononuclear cell inflammation indicative of environmental antigenic stimulation were observed in some of mock-infected control mice. No other abnormalities were detected in mock-infected mice.

Interestingly, at 4 dpi, mice treated with 9F4-LALA presented similar pathology as the 1A4-treated mice despite ∼17-fold reduction in viral load. Minimal tissue damage, suppurative and mononuclear cell inflammation were present in the lungs of 9F4-WT treated mice, suggesting that 9F4-WT is much more effective than 9F4-LALA in reducing virus pathogenesis (Figure 4D and E).

By 14 dpi, all the 9F4-WT treated and 75% of 9F4-LALA treated mice have regained 100% of body weight prior to H5N6 infection. Consistently, both 9F4-WT and 9F4-LALA treated mice at 14 dpi exhibited no signs of tissue degeneration suggesting lung recovery and successful treatment of IAV. Mild and marked levels of mononuclear cell inflammation were present respectively in the lungs of 9F4-WT and 9F4-LALA treated mice 14 dpi (Figure 4D and E). Presence of chronic lung inflammation at 14 dpi is likely explained by the recruitment and proliferation of mononuclear cells due to antigenic stimulation from IAV during the infection phase.

### CDC is dispensable for the *in vivo* protective function of 9F4

To assess if the CDC pathway plays a major role in 9F4’s anti-H5N6 activity, mice intranasally infected with 100 PFU of rgPR8 H5N6 were treated intraperitoneally 24 hpi with 10 mg/kg of 9F4-WT, 9F4-K322A or 1A4. Infected mice that received 1A4 lost weight steadily (Figure 5A) and died at 8 dpi (Figure 5B). In contrast, infected mice treated with 9F4-K322A lost approximately 5% body weight between 1 and 3 dpi but maintained normal body weight profiles similar to that of mock-infected mice or infected mice that received 9F4-WT (Figure 5A). All infected mice that were treated with 9F4-WT or 9F4-K322A survived the rgPR8 H5N6 challenge (Figure 5B). 9F4-WT and 9F4-K322A treated mice had similar lung virus titres at 4 dpi and were both significantly lower compared to mice treated with 1A4 (Figure 5C).

Analyses of mice lungs at 4 dpi showed consistent findings as previously observed (Figure 4D and E) in that 9F4-WT treated mice showed reduced damage of the bronchial and alveolar epithelium, tissue necrosis and polymorphonuclear cell inflammation compared to infected mice treated with 1A4. 9F4-K322A treated mice also showed reduced tissue damage and polymorphonuclear cell inflammation compared to 1A4 treated mice, albeit to a lesser extent compared to mice that received 9F4-WT (Figure 5D and E). Collectively these observations suggest that 9F4-K322A has similar anti-H5N6 potency compared to 9F4-WT and that CDC is dispensable for 9F4’s capability to protect against H5N6 *in vivo*.

### Alveolar macrophages are crucial for 9F4-WT’s antiviral activity

The introduction of LALA and K322A mutations to the Fc region of 9F4 have allowed us to study the importance ADCC/ADCP or CDC respectively in the context of 9F4’s antiviral activity. However, mutations that will allow specific inhibition of ADCP but not the other Fc mediated pathways have not yet been identified. We therefore opted to study the importance of alveolar macrophages, which are the main effectors of phagocytosis, in the context of 9F4 protection against H5N6. To deplete lung phagocytes, mice were intranasally given clodronate encapsulated liposomes, two days before and during virus infection with 100 PFU of rgPR8 H5N6, followed by a single 10 mg/kg dose of 9F4-WT administered intraperitoneally at 1 dpi. Control mice were given control liposomes containing PBS. Flow cytometric analyses of total lung mononuclear fractions from uninfected mice showed reduction of alveolar macrophages by at least 90% up to 6 days after clodronate liposomes were given (Supplementary Figure S2).

**Figure 6.**
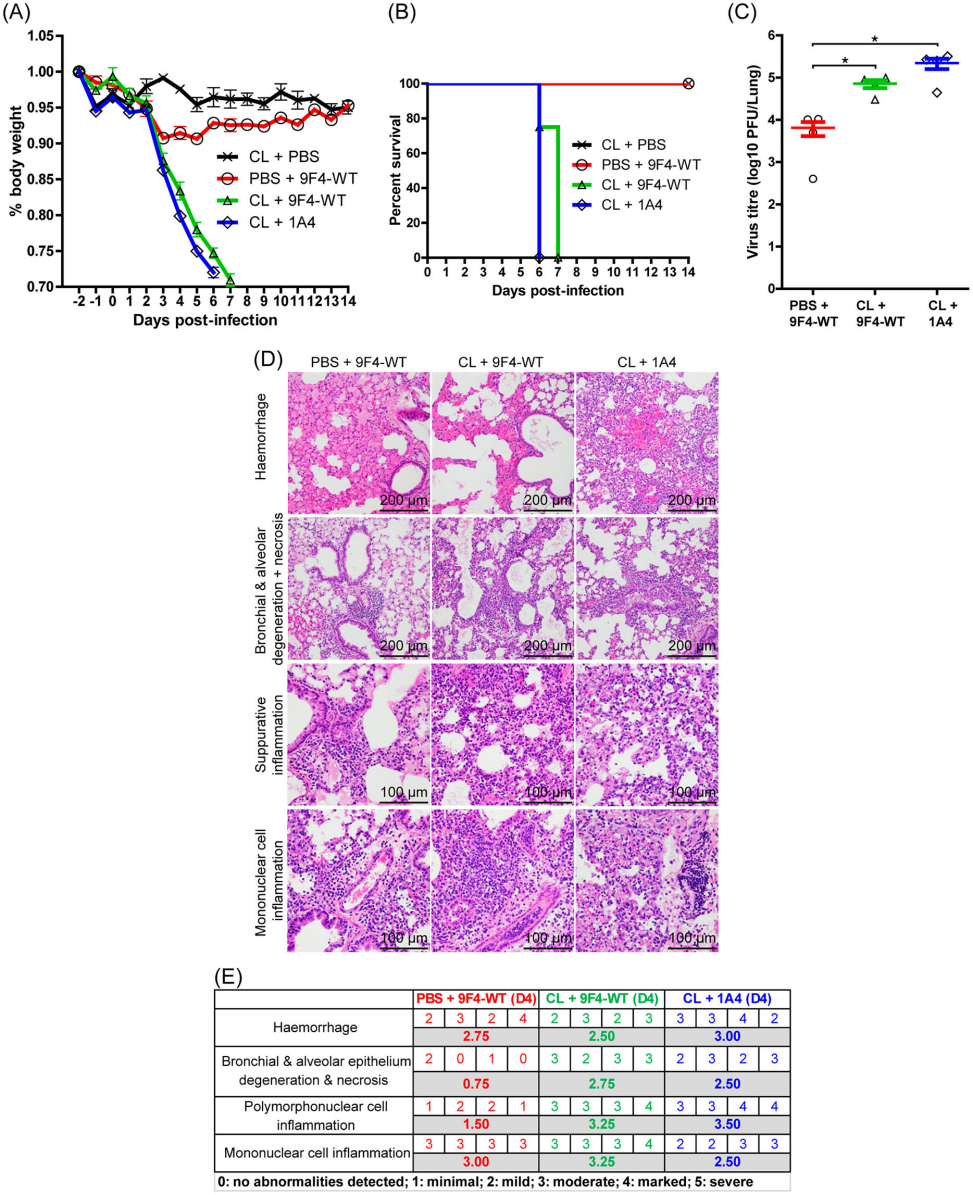
Alveolar macrophages are crucial for *in vivo* protection against H5N6 IAV infection by 9F4-WT. Balb/c mice were intranasally given 20 µl of clodronate encapsulated liposomes (CL) or control liposomes (PBS) 2 days prior and simultaneously during infection with 100 PFU of rgPR8 H5N6. Infected mice received a single therapeutic dose of 9F4-WT or the isotype control 1A4 at 10 mg/kg whereas mock-infected mice received an equivalent volume of PBS via intraperitoneal injections 24 hpi. (A) Body weight profiles and (B) animal survival are shown. (C) Lung virus titres were determined at 4 dpi by plaque assays with MDCK cells. (D) Representative images of H&E stained formalin fixed paraffin embedded lung sections along with their (E) corresponding histopathological grades of individual mice and averages of treatment groups at 4 dpi. *n* = 4 per treatment group. Error bars represent SEM, asterisks indicate *p* < 0.05.

Uninfected mice that received clodronate liposomes (CL + PBS) lost approximately 5% body weight within 2 days after mock-infections but maintained >95% original body weight throughout the study (Figure 6A) with a 100% survival rate (Figure 6B). Mice that received control PBS encapsulated liposomes, infected with rgPR8 H5N6 and treated with 9F4-WT (PBS + 9F4-WT) lost up to 10% body weight by 5 dpi but started to regain body weight between 6 and 14 dpi (Figure 6A) and survived the H5N6 challenge (Figure 6B). In contrast, clodronate liposome treated mice that were infected and received a therapeutic dose of 9F4-WT (CL + 9F4-WT) or the irrelevant antibody 1A4 (CL + 1A4) lost weight steadily from 3 dpi (Figure 6A) and died by 7 and 6 dpi respectively (Figure 6B). At 4 dpi, significant reductions of virus titres were detected in PBS + 9F4-WT mice (6.5 × 10^3^ PFU/lung) compared to both CL + 9F4-WT (7.2 × 10^4^ PFU/lung) and CL + 1A4 (2.2 × 10^5^ PFU/lung) mice (Figure 6C), demonstrating the importance of alveolar macrophages in 9F4’s antiviral activity.

Histology analyses of mice lungs at 4 dpi reveal similar moderate levels of haemorrhage and mononuclear cell inflammation in all 3 groups of mice (Figure 6D and E). Increased levels of lung mononuclear cell inflammation detected in mice that received any form of liposomal treatment (Figure 6D and E) compared to those that did not (Figure 4D and E) suggests that mononuclear cell inflammation was caused by the presence of liposomes. PBS + 9F4-WT mice displayed minimal bronchial and alveolar epithelial degeneration and mild levels of suppurative inflammation. In contrast, infected mice depleted of alveolar macrophages (CL + 9F4-WT and CL + 1A4) had moderate levels of bronchial and alveolar epithelial degeneration and suppurative inflammation regardless of which antibody was given therapeutically. Collectively, the body weight profiles, mice survival rates, lung virus titres and histopathology results demonstrate that alveolar macrophages are crucial for 9F4’s antiviral activity.

### Localized administration by intranasal instillation of 9F4-WT also protects against H5N6 infection

**Figure 7.**
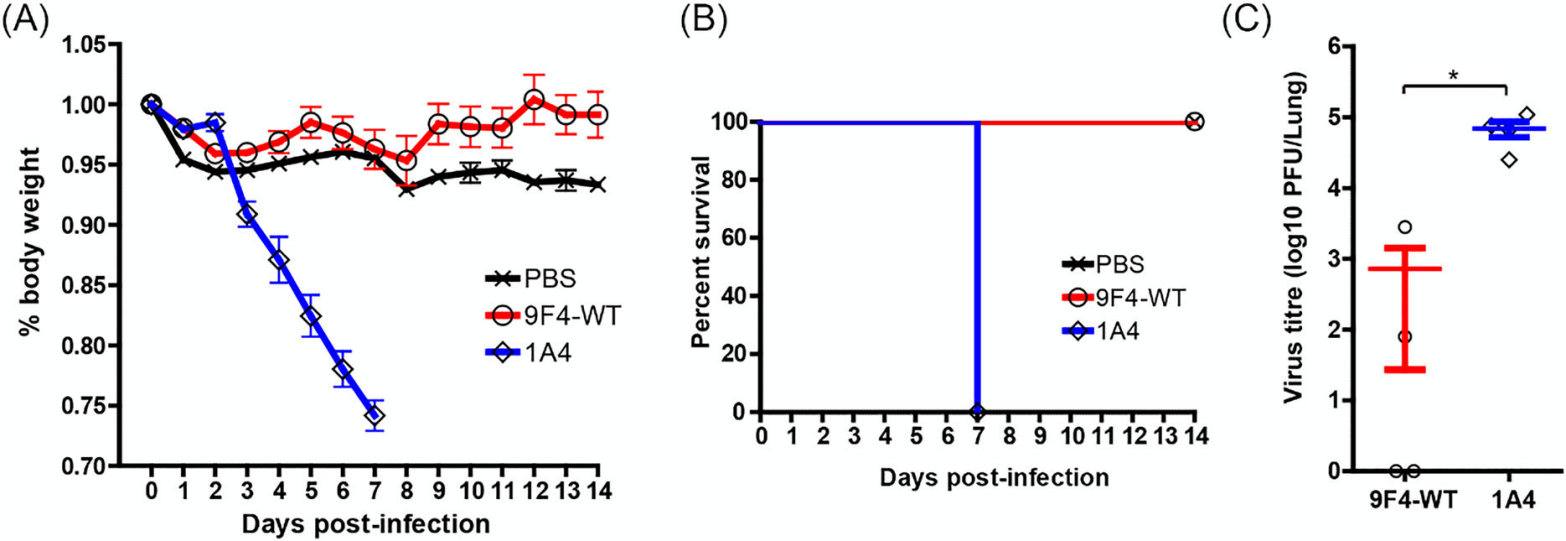
Intranasal administration of 9F4-WT is able to protect against H5N6 challenge *in vivo*. Balb/c mice were infected with 100 PFU of rgPR8 H5N6 and received a single therapeutic dose of 9F4-WT, 9F4-LALA or the isotype control 1A4 at 2 mg/kg via intranasal instillations 24 hpi. (A) Weight loss profiles and (B) animal survival are shown. (C) Lung virus titres were determined 4 dpi by plaque assays with MDCK cells. *n* = 4 per treatment group, error bars represent SEM, asterisks indicate *p* < 0.05.

Studies have shown the successful treatment of mice challenged with various influenza subtypes by intranasal delivery of antibodies [44,55–57] or antibody encoding adeno-associated virus vectors [58,59]. Furthermore, it was recently established that localized administration of antibodies via intranasal instillation or aerosolisation requires a lower dose of anti-stalk antibodies for protection against influenza infections [44]. To determine if this observation also applies to the VE targeting antibody 9F4, infected mice were intranasally treated with a lower dose of 2 mg/kg of 9F4-WT, 24 hpi (Figure 7). Infected mice intranasally treated with 1A4 (irrelevant control antibody) showed similar weight loss profiles and lung virus titres (6.93 × 10^4^ PFU/lung; Figure 7A and C) compared to those that received 1A4 intraperitoneally (Figure 4A and C). All infected mice receiving 1A4 intranasally died at 7 dpi (Figure 7B). Contrary to 1A4 treated mice, all of the infected mice that received a 2 mg/kg dose of 9F4-WT intranasally survived the H5N6 infections, had similar weight loss profiles compared to mock-infected mice and significantly lower lung virus titres (7.20 × 10^2^ PFU/lung) compared to 1A4 treated mice (Figure 7C). These findings are consistent with the view that localized administration of therapeutic antibodies increases their efficacies against influenza infections [44,55].

## Discussion

9F4 was previously characterized to bind to the VE domain within the HA protein of H5N1 [38]. Similar to the characteristics of other VE binding antibodies that recognize a range of different epitopes within the VE domain [34–37], 9F4 was able to bind to different clades of H5 IAV. In this study, we show that 9F4-WT, 9F4-LALA and 9F4-K322A bind and neutralize the reassortant HPAI H5N6 strain both *in vitro* and *in vivo*. The ability of 9F4 to confer protection in mice challenged with rgPR8 H5N6 demonstrates the utility of 9F4 as a therapeutic against H5 outbreaks. The ability of another VE targeting mAb, 100F4, to neutralize various H5 subtypes has also been previously reported [35]. Collectively these findings indicate that the VE domain is relatively conserved between strains of the same virus subtype and can be used effectively as a target for influenza therapy.

In addition to 9F4’s known function to inhibit virus entry, the Fc-mediated response component of 9F4 was investigated in this study. Our results show that 9F4-LALA, a ADCC and ADCP deficient mutant of 9F4, was able to confer protection in rgPR8 H5N6 challenged mice. This observation indicates that inhibition of pH-mediated membrane fusion by 9F4 was sufficient for protection against H5N6 infections in mice with intact immune systems. However, a comparison of 9F4-WT and 9F4-LALA shows that ADCC and/or ADCP contributes significantly to 9F4’s antiviral activity given that 9F4-WT treatment outperforms 9F4-LALA in all aspects examined in this study such as mice survival rates, degree of weight loss, lung virus titres and pathology outcomes (Figure 4). On the contrary, the observation that 9F4-K322A had similar antiviral efficacy compared to 9F4-WT indicates that CDC does not play a major role in 9F4’s antiviral function (Figure 5). Our results suggest that the ADCC and/or ADCP pathways are partially responsible for 9F4’s antiviral potency and concurs with another study demonstrating that a D265A ADCC deficient mutant of the VE-targeting antibody 1H5 was able to protect mice from H7 infections but is less potent compared to wild type 1H5 [36].

Since alveolar macrophages are known mediate ADCP in IAV infections [41,60] directly via phagocytosis and indirectly by secretion of various cytokines, we attempted to further dissect the ADCC and ADCP pathways in 9F4 function by investigating 9F4-WT’s anti-H5N6 activity in mice that were depleted of alveolar macrophages. Interestingly, 9F4 was unable to protect depleted mice against H5N6 challenge (Figure 6), suggesting that 9F4’s anti-H5N6 activity *in vivo* likely requires the presence of alveolar macrophages.

Although 75% of rgPR8 H5N6 infected mice treated with 9F4-LALA survived and had an approximate 17-fold lung virus titre reduction compared to mice treated with an irrelevant isotype control 1A4 (Figure 4C), the lungs of 9F4-LALA treated mice at 4 dpi showed similar degrees of tissue injury and inflammation when compared to 1A4 treated mice (Figure 4D and E). This suggests that clearance of the virus particles promptly, potentially via ADCC or ADCP may be important for the control of lung tissue damage and inflammation.

A recent study [44] demonstrated that localized administration of a stalk binding antibody to mice lungs via aerosol inhalation or intranasal instillation increased their therapeutic efficacy when compared to those administered via systemic routes. Our results that intranasal instillation of 9F4-WT was also protective against H5N6 infections demonstrate that VE targeting antibodies were also effective via localized administration (Figure 7). Furthermore, the observation that 9F4-WT was effective against H5N6 when given intranasally at a lower dose of 2 mg/kg supports the notion that localized administration of IAV antibodies to the lung is more efficacious compared to the systemic administration routes. It would be interesting to assess if intranasal administration of anti-IAV therapeutics would also be effective in the ferret model since IAV infected ferrets exhibit human like clinical symptoms such as sneezing and nasal discharge, which may complicate intranasal or aerosol based therapies.

## Supplementary Material

Supplemental Material
