# Supplementary material for: Contribution of Fc-dependent cell-mediated activity of a vestigial esterase-targeting antibody against H5N6 virus infection
Source: Emerg Microbes Infect. 2020 Jan 6;9(1):95–110. doi: 10.1080/22221751.2019.1708215 (PMC6968706; doi:10.1080/22221751.2019.1708215)
Supplement: Supplemental Material [file TEMI_A_1708215_SM6941.zip › Supplementary Material.docx]

**Supplementary Figure S1. 9F4 does not bind to other virus subtypes.** (**A**) Alignment of residues 50 to 80 in the HA protein of H5N1, H3N2, H7N7, H7N9, H9N2 viruses. For comparison, H3 numbering is followed. The residues which are important for the interaction with 9F4, namely R62, W69 and F79, are boxed. (**B**) ELISA was performed to determine the ability of 9F4 to bind to the HA of different subtypes. Wells were coated with HA proteins of H5Nx (H5N1 (A/Vietnam/1194/2004); H5N2 (A/chicken/Iowa/04-20/2015); H5N6 (A/duck/Guangdong/GD01/2014); H5N8 (A/broiler duck/Korea/Buan2/2014)) and incubated with serially diluted 9F4. 1A4 was also used at the highest concentration (50 ng/ml) as an isotype control antibody.

**Supplementary Figure S2. Depletion of alveolar macrophages by clodronate liposome.** Balb/c mice received two 20 or 50 µl doses of clodronate encapsulated (CL) or two 50 µl doses of control (PBS) liposomes at day 0 and day 2. Mice were sacrificed on day 8 and mononuclear cells were purified from lung homogenates and analysed via flow cytometry for the presence of eosinophils (CD45.2^+^ Mac1^+^ Siglec F^+^), interstitial macrophages (IM: CD45.2^+^ Mac1^+^ Siglec F^-^ CD11c^+/-^) and alveolar macrophages (AM: CD45.2^+^ Mac1^-^, Siglec F^+^ CD11c^+^). (**A**) Gating strategy for identification of eosinophils, IM and AM. (**B**) Proportions of DAPI^-^ CD45.2^+^ cells corresponding to eosinophils, IM and AM in mice treated with 2x 50 µl doses of control PBS liposomes, 2x 20 µl or 2x 50 µl doses of clodronate liposomes. Representative plots are shown. (**C**) Absolute number of eosinophils, IM, AM and the percentage decrease in AM relative to PBS liposome control.
