# Supplementary figures and images for: Contribution of Fc-dependent cell-mediated activity of a vestigial esterase-targeting antibody against H5N6 virus infection
Source: Emerg Microbes Infect. 2020 Jan 6;9(1):95–110. doi: 10.1080/22221751.2019.1708215 (PMC6968706; doi:10.1080/22221751.2019.1708215)

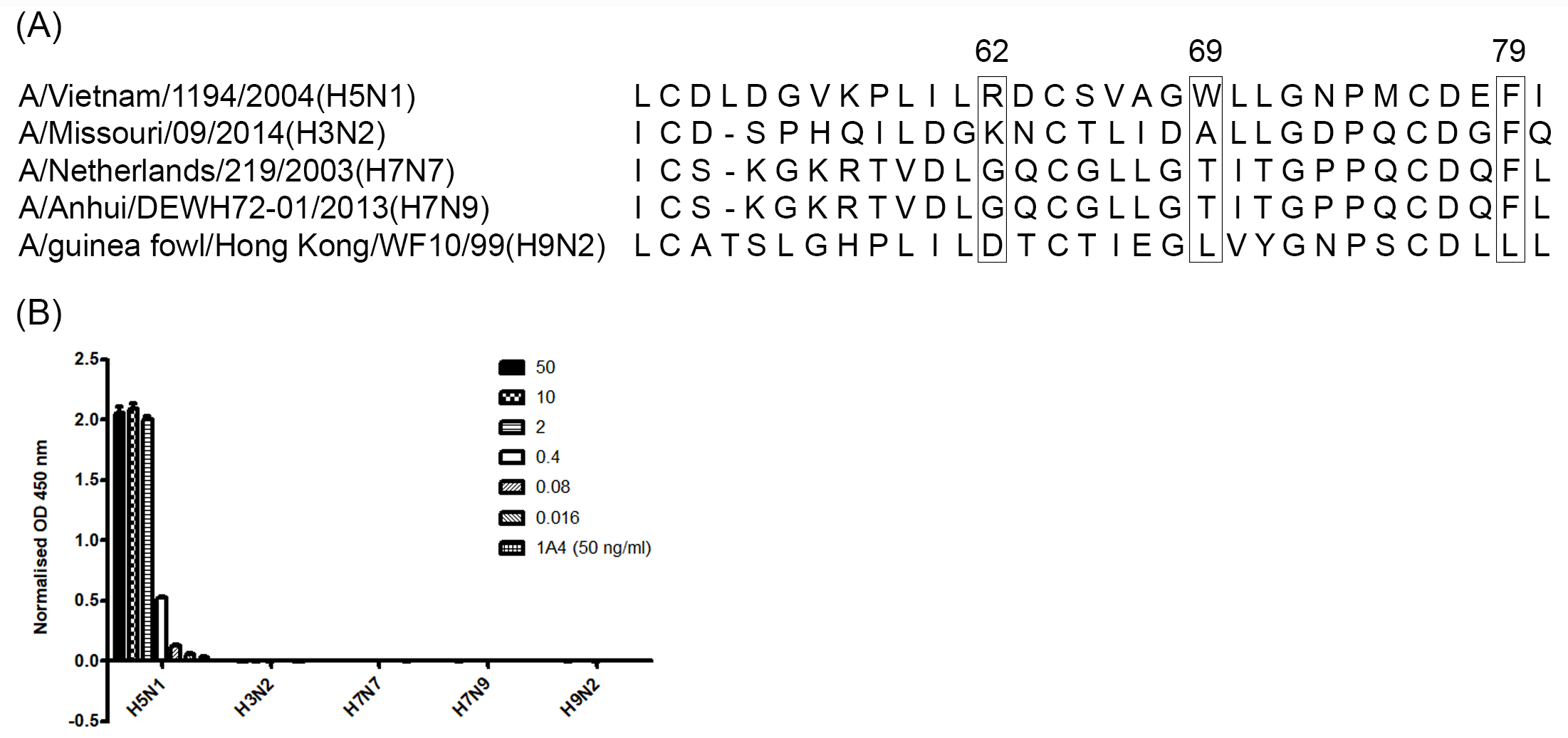

Supplement: Supplemental Material [file TEMI_A_1708215_SM6941.zip › Supplementary Fig S1.tif]

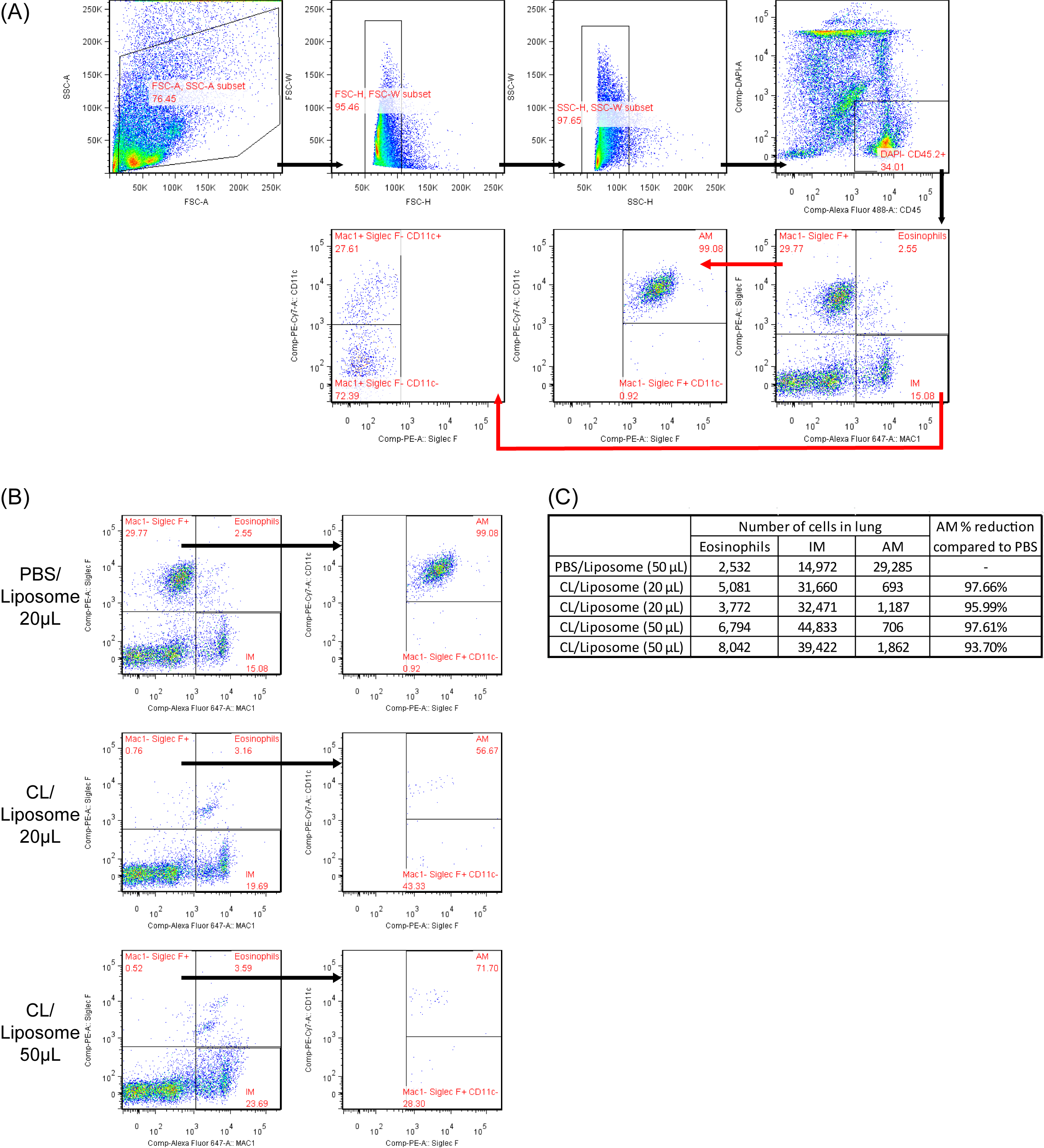

Supplement: Supplemental Material [file TEMI_A_1708215_SM6941.zip › Supplementary Fig S2.tif]
